# Identification of transcripts involved in meiosis and follicle formation during ovine ovary development

**DOI:** 10.1186/1471-2164-9-436

**Published:** 2008-09-23

**Authors:** Adrienne Baillet, Béatrice Mandon-Pépin, Cédric Cabau, Elodie Poumerol, Eric Pailhoux, Corinne Cotinot

**Affiliations:** 1 INRA, ENVA, UMR 1198 Biologie du Développement et Reproduction, F-78350 Jouy en Josas, France; 2 CNRS, FRE 2857, F-78350 Jouy en Josas, France; 3 INRA, SIGENAE, UR83 Unité de Recherches Avicoles, 37380 Nouzilly, France

## Abstract

**Background:**

The key steps in germ cell survival during ovarian development are the entry into meiosis of oogonies and the formation of primordial follicles, which then determine the reproductive lifespan of the ovary. In sheep, these steps occur during fetal life, between 55 and 80 days of gestation, respectively. The aim of this study was to identify differentially expressed ovarian genes during prophase I meiosis and early folliculogenesis in sheep.

**Results:**

In order to elucidate the molecular events associated with early ovarian differentiation, we generated two ovary stage-specific subtracted cDNA libraries using SSH. Large-scale sequencing of these SSH libraries identified 6,080 ESTs representing 2,535 contigs. Clustering and assembly of these ESTs resulted in a total of 2,101 unique sequences depicted in 1,305 singleton (62.11%) and 796 contigs (37.9%) ESTs (clusters). BLASTX evaluation indicated that 99% of the ESTs were homologous to various known genes/proteins in a broad range of organisms, especially ovine, bovine and human species. The remaining 1% which exhibited any homology to known gene sequences was considered as novel. Detailed study of the expression patterns of some of these genes using RT-PCR revealed new promising candidates for ovary differentiation genes in sheep.

**Conclusion:**

We showed that the SSH approach was relevant to determining new mammalian genes which might be involved in oogenesis and early follicle development, and enabled the discovery of new potential oocyte and granulosa cell markers for future studies. These genes may have significant implications regarding our understanding of ovarian function in molecular terms, and for the development of innovative strategies to both promote and control fertility.

## Background

Differentiation of the mammalian gonad is a dynamic process which occurs during embryogenesis and continues throughout an individual's life. During this process, both germ and somatic cells acquire sex-specific characteristics. Meiosis is initiated at different time points in males and females; female germ cells begin meiosis during embryogenesis while male germ cells do not enter meiosis until puberty. Recent findings have indicated that the entry of female germ cells into meiosis is mediated by retinoid responsive genes [1,2]. A second major event in female gonad differentiation occurs when somatic pre-granulosa cells surround individual germ cells to form primordial follicles which constitute a reservoir of oocytes [3], available for subsequent growth and differentiation. This event occurs during fetal life or after birth, depending on the species.

During the last decade, substantial progress has been made in identifying the genes that control the assembly and initial growth of ovarian follicles. Different approaches, such as transcriptome analysis of fetal ovaries at different developmental stages and gene inactivation in the mouse, have revealed pathways leading to the formation and differentiation of follicles [4-10]. The transcription of several oocyte-specific genes, such as growth and differentiation factor 9 (*Gdf9*) and bone morphogenetic protein 15 (*Bmp15*) commences during early folliculogenesis [11-13]. Regulation of these genes is in part due to the expression of two oocyte-specific transcription factors, *Figla *[5] and *Nobox *[8]. It has been also shown recently that *Sohlh1 *disruption perturbs follicular formation, partly by causing the down-regulation of *Nobox *and *Figla *[6].

In order to identify novel genes involved in follicle differentiation, microarray-generated expression profiles of the mature ovary have been compared with those of newborns, and several novel genes in ovarian development and follicle formation have thus been discovered [14-16].

By contrast, few genes are known to be related to meiotic prophase I in female mammals. In most cases, meiotic genes were initially discovered in the context of meiotic recombination in yeast. Mammalian counterparts were then discovered, predominantly by means of approaches such as gene-targeting in mice and mutational and chromosomal analysis in human patients with fertility disorders [17-24]. The application of microarray technology has enabled the comparison of transcriptional activity in developing male and female gonads at different stages, some of which contain female germ cells in meiotic prophase I [25-27]. More recently, a microarray study compared genome-wide expression in embryonic mouse ovaries, the aim being to identify novel genes associated with early female meiosis, and reported that around 200 genes were differentially expressed in E13.5 mouse ovaries compared with E11.5 ovaries [28].

Another means of isolating differentially expressed transcripts consists in using subtraction techniques. A highly effective method, termed suppression subtractive hybridization (SSH), has been applied to generating subtracted cDNA libraries of various systems. This method has also been used to isolate ovary-specific genes in several species [29-32].

In sheep, differences between the somatic components of ovaries and testes are microscopically evident by embryonic day 35 (D35). Germ cells, however, remain indistinguishable between the sexes until D55, when ovarian germ cells initiate prophase of meiosis (between days 49 and 56) and testicular germ cells arrest in the *G*_0_/G_1 _stage of the mitotic cell cycle [33]. The structural integrity of ovigerous cords is maintained until D75, at which time primordial follicles first begin to emerge from the interface of the cortex and medulla [34]. The maximum number of germ cells present within the fetal ovary of sheep is found at D75 (i.e., 800,000). Type 2 follicles (an oocyte with at least one but not two complete layers of cuboidal granulosa cells) are first observed at around D90, and in some cases, type 3 (an oocyte with 2 to <4 layers of cuboidal granulosa cells) and type 4 (an oocyte with 4 to >6 layers of cuboidal granulosa cells with no evidence of antrum formation) follicles are present by D120. Antral follicles are observed in the fetal sheep ovary at around D135, which is about 12 days before parturition [35]. Few studies have described the expression profiles of genes controlling meiosis and follicular development in sheep [36-40].

In this study, we report on the generation of two sheep fetal ovary cDNA libraries, obtained by applying SSH using mRNAs isolated from D55 and D82 female gonads In the first subtraction D55 was used as tester and D82 as driver and the forward subtractive library was constructed (M, Meiosis). In the second one the tester and driver were interchanged and the reverse subtractive library was constructed (F, Folliculogenesis). These two stages correspond to the onset of meiotic prophase I in female germ cells and primordial follicle formation, respectively. 7,296 cDNA clones were selected and 6,080 were sequenced from both libraries, Then 5,025 valid sequences were subjected to database searches to annotate the putative functions of the genes thus represented. Differentially expressed genes were identified by in silico analysis and RT-PCR. Expression analysis confirmed that some of these genes were specifically expressed in fetal ovaries. We further demonstrated that two of the selected ESTs [EMBL:CU651916 and EMBL:CU654655] were expressed in the female gonad during meoitic prophase I period. Furthermore, about 1% (21/2101) of these ovary-expressing transcripts were novel. They were analyzed for their expression in gonads at different developmental stages. The expression patterns of these 21 cDNAs were validated by RT-PCR, which enabled the identification of two transcripts with ovary-specific expression.

## Results

### Generation of two ovary stage-specific cDNA libraries

Messenger RNAs isolated from D55 and D82 fetal ovaries were used to construct two subtractive cDNA libraries using SSH. The M (meiosis) library, supposed to be enriched in cDNAs representing genes preferentially expressed during prophase I meiosis, was obtained using cDNAs from D55 mRNAs as the 'tester' and cDNAs from D82 mRNAs as the 'driver'. The F (folliculogenesis) library was obtained by reversing the 'tester' and 'driver' mRNA. Sequencing was carried out for 6,080 cDNAs clones selected at random from both libraries: 3,203 from the M library and 2,877 from the F library. After removing poor quality sequences, a total of 2,474 ESTs from the M library and 2,551 from the F library were conserved for further analyses (Table 1). The insert size of the clones ranged from 99 to 1362 base pairs, and the average insert size was approximately 450 base pairs.

**Table 1:** Summary of the ESTs generated from two ovine SSH libraries.

	**Meiosis Library**	**Folliculogenesis Library**	**TOTAL**
Number of clones in each library	3456	3840	7296
Number of clones sequenced in each library	3203	2877	6080
Number of clones valid after sequencing	2474	2551	5025
Number of clones not valid after sequencing	729	326	1055
Number of contigs	1578	957	2535
Number of uncommon contigs	1361	740	2101
Number of contigs with UniGene annotation	1346	734	2080
	98.8%	99%	
Number of contigs with Swiss-Prot annotation	769	404	1173
	56.5%	54.8%	
Number of contigs with GO annotation	344	199	543
	25.3%	26.8%	
Number of contigs without any annotation (unknown)	15	6	21
	1.1%	0.8%	

**Figure 1:**
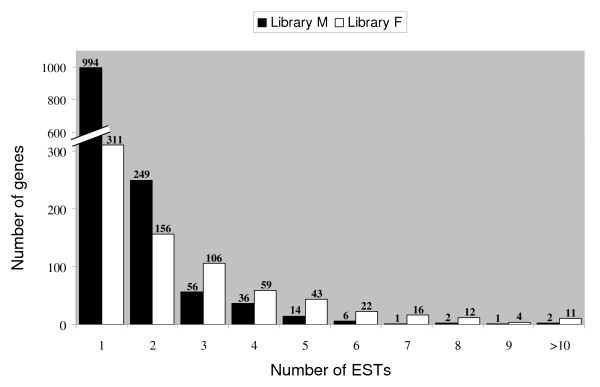
**Distribution and numbering of assembled sequences**. Histogram of gene representation after the suppressive subtractive hybridization (SSH) procedure. Most genes were present only once in the library (62%); genes present once or twice represented 81.4% of the clones, emphasizing the normalizing effect of the method.

A database named M/F db was generated using all ESTs from the M and F libraries [41]. To generate contigs regrouping identical ESTs, two successive criteria were applied. The first step built clusters of all 5,025 sequences from the M/F db and publicly available ovine ESTs, sharing at least 75 bp at an identity rate of 96% using MegaBlast. The second step constructed coherent contigs from the previous clusters using the CAP3 assembly program [42]. This analysis revealed a total of 2,101 independent contigs (82.8% of the total number of contigs) for both libraries, 1,361 contigs only being found in the M library and 740 in the F library (Table 1). These comparisons resulted in the assembly of 2,101 independent sequences including 796 clusters (contigs) and 1,305 singleton ESTs (Fig. 1). The percentage of unique sequences in each library ranged from 42% (F) to 73% (M). This high proportion of sequences represented by single ESTs seemed to indicate an efficient normalization of the libraries; only 3.7% of the genes were represented by more than 5 ESTs. Furthermore, only 8.5% of the sequences were represented by ESTs from both libraries (Table 1), as expected for libraries that were indeed normalized.

### Sequence analysis

**Figure 2:**
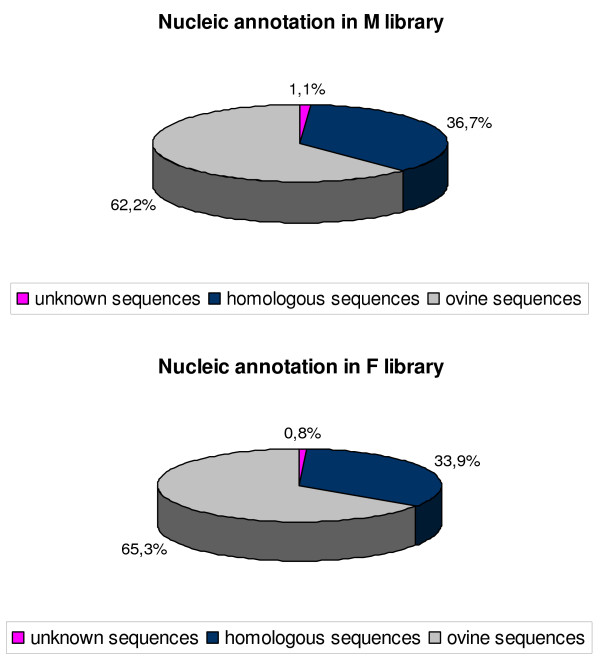
**Sequence analysis of the two SSH libraries**. The M library contained 847 contigs or unique sequences with an ovine UniGene annotation (Ovis aries: UniGene Build #11 database) + 491 contigs or unique sequences with a bovine or human UniGene annotation (Bos taurus: UniGene Build #84, Homo sapiens: UniGene Build #202 databases) + 15 sequences without gene annotation. The F library comprised 483 contigs or unique sequences with an ovine UniGene annotation (Ovis aries: UniGene Build #11 database) + 248 contigs or unique sequences with bovine or human UniGene annotation (Bos taurus: UniGene Build #84, Homo sapiens: UniGene Build #202 database + 6 sequences without gene annotation.

Each sequenced clone was analyzed using the BLAST program and classified as known, homologous, or novel after its evaluation against entries within the publicly accessible non-redundant NCBI database (Fig. 2). Known genes were classified as such if homology was high (identity of 90% or more) between the subtracted SSH-generated clones and the Ovis aries database: UniGene Build #11. They accounted for more than 60% in both libraries (62.2% and 65.3% in the M and F libraries, respectively).

Contigs were classified as homologous sequences if they possessed homology with bovine or human gene sequences, relative to entries in the Bos taurus: UniGene Build #84, Homo sapiens: UniGene Build #202 NCBI database. Homologous contigs accounted for 36.7% in the M library and 33.9% in the F library. Novel ESTs were designated as such if no significant match was obtained in the different databases with known genes (Fig. 2). Fifteen were found without gene annotation in the M library (15/1361, 1.1%) and six in the F library (6/740, 0.8%). The two most represented genes in the M library were Cytochrome C oxidase subunit 3 (EC 1.9.3.1) and Bos taurus hypothetical protein, LOC783526, with 13 and 12 ESTs respectively. Eleven genes were found more than 10 times in the F library [see Additional file 1].

### Functional annotation of ESTs

**Figure 3:**
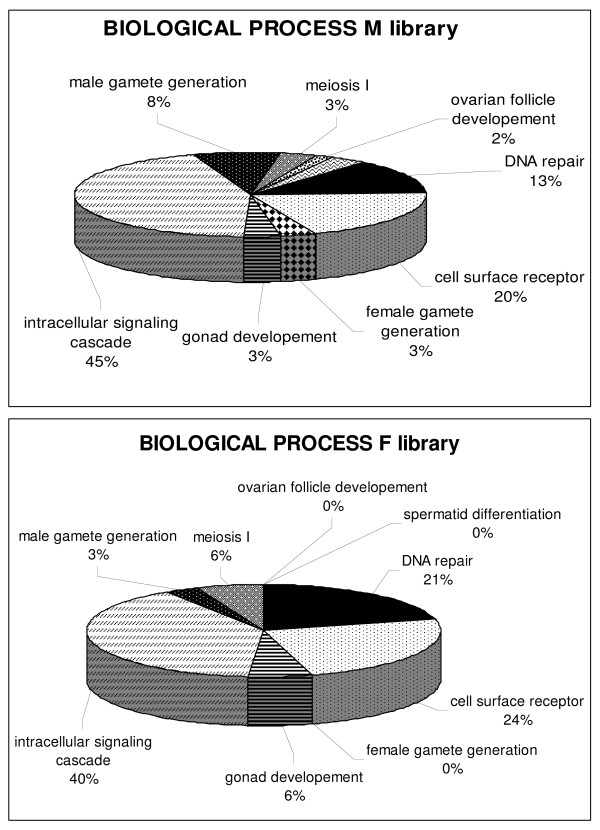
**Percentage representation of gene ontology (GO) mappings for the two SSH libraries**. Functional annotation was performed using the WEGO (Web Gene Ontology Annotation Plot), a valuable tool for the plotting of GO annotation results. The pie diagrams show the distribution of 543 sequences in the "biological process" GO category.

Comparison of the 2,101 sequences from both SSH libraries with the non-redundant protein UniProt/SwissProt database revealed that 1,173 contigs (55.8%) displayed significant similarity (<1E-5) to existing protein sequences. Furthermore, among these 2,101 contigs, only 543 had one GO term (25.8%). These 543 sequences fell into the three principal GO categories: cellular component, biological process and molecular function. In both libraries, the three most widely represented biological processes were intracellular signaling cascade (40–45%), cell surface receptor (20–24%) and DNA repair (13–21%) (Fig. 3). However, the small number of GO annotated sequences restricted functional annotation of our libraries.

### Identification of differentially expressed ovary-specific transcripts

In order to evaluate the specificity of the transcripts involved in both the developmental processes studied here, i.e. meiosis and follicle formation, individual library specific ESTs were studied. A bioinformatics comparison of our SSH library sequences with those published in the literature showed that at least 10 genes previously described as being expressed in mice fetal ovaries or adult male meiotic germ cells were present in our libraries. The detection of several genes already known to be expressed during meiosis, such as *Stk31 *[EMBL:CU655270], *Smc3 *[EMBL:CU653776], *Dmrt7 *[EMBL:CU652558], *Zp148 *[EMBL:CU655301], *Mutl1 *[EMBL:CU654415], *Dazl *[EMBL:CU654830], *Stathmin *[EMBL:CU652110], *Msh2 *[EMBL:CU654850] and *Msh5 *[EMBL:CU654104] in the M library increased confidence in the quality of the subtraction procedure. In a similar manner, sequences corresponding to the *Foxo3A *[EMBL:CU638379], *Lhx8 *[EMBL:CU637755] and *BMP4 *[EMBL:CU653220] genes involved in folliculogenesis were present in the F library.

**Figure 4:**
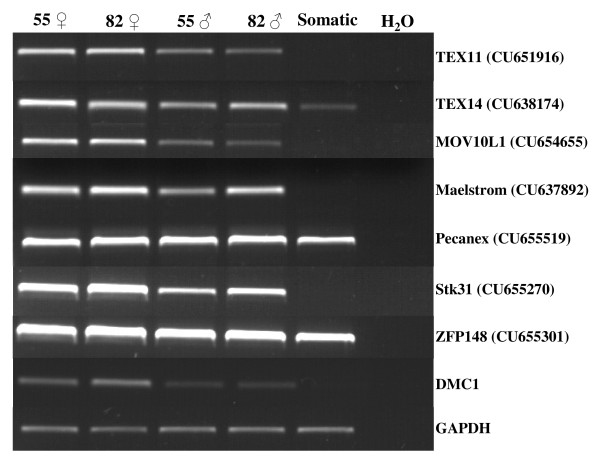
**Mini profiles of transcripts selected in the M library**. Semi-quantitative RT-PCR analysis of seven selected genes from the M library and the meiosis gene DMC1 in cDNAs from male and female gonads at 55 and 82 days post coitum. Somatic samples correspond to a mix of brain, kidney, liver and heart obtained from Day 55 fetuses. *Pecanex 1 *and *ZFP148 *transcripts were present in all tested samples. Only *TEX11, MOV10L1, STK31 *genes were preferentially expressed in ovaries, presenting an expression pattern similar to *DMC1*. *GAPDH *(glyceraldehyde-3P-dehydrogenase) was used as a control for the concentration of cDNA populations. ♀: female and ♂: male.

**Figure 5:**
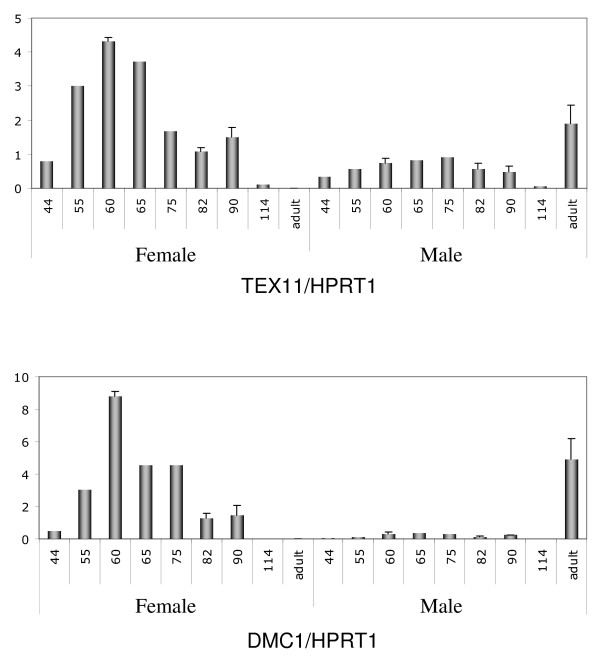
**Real-time quantitative RT-PCR expression of meiosis-specific transcripts**. Quantification of TEX11 and DMC1 gene expression in sheep ovaries at the stages indicated (D44, 55, 60, 65, 75, 82, 90, 114 and adult). HPRT1 (hypoxanthine phosphoribosyltransferase) gene was used as a reporter gene. For both genes, a peak was observed at around 60 dpc then a decrease as from 75 dpc. No expression was detected in adult ovary. In males, levels of transcription were very low during fetal life. By contrast, strong expression was observed in adult testis. SEM were calculated at stages D60, 82, 90 and adult.

RT-PCR screening was used to validate in our animal model the ovary-specific expression of several genes previously described in the context of mouse gonad transcriptome analysis. This screening contained (i) a mixture of cDNAs from four different fetal somatic tissues (kidney, liver, heart and brain) and (ii) cDNA from male and female gonads at two different developmental stages (D55 and D82). We examined seven genes from the M library using this mini-profiling procedure, with *Dmc1 *as the positive control of the meiotic gene (Fig. 4). Five genes (*TEX11*, *TEX14*, *MOV10L1*, *Maelstrom, STK31*) produced a band-pattern consistent with the profile expected for a gonad-specific transcript or a predominantly gonad-expressed transcript. Three of them (*TEX11*, *MOV10L1*, *STK31*) were more strongly expressed in female gonads than in males (Fig. 4). In order to confirm their specific expression pattern, TEX11 was subjected to further real-time quantitative RT-PCR expression analysis on a panel of several fetal stages for each sex. Its expression profile appeared to be specific to meiotic prophase I and was similar to typical meiotic genes such as *DMC1 *(Fig. 5).

**Figure 6:**
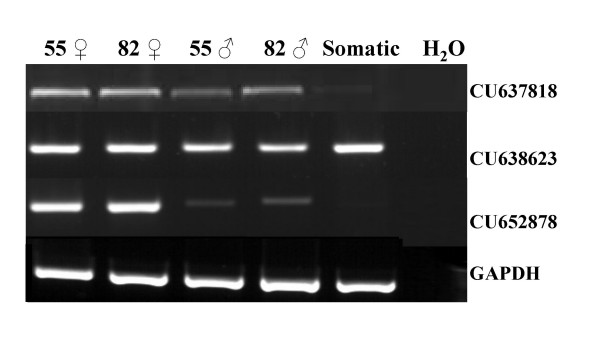
**Mini profiles of unknown transcripts**. Results from semi-quantitative RT-PCR analyses showing the expression of three representative ESTs from the 21 unknown transcripts at two stages (Days 55 and 82) of male and female gonad development; GAPDH served as an internal control to monitor the amount of RNA. The 21 ESTs are divided into three categories: those having a gonad-specific pattern such as CU637818, those expressed in gonads and somatic tissues represented here by CU638623, and those differentially expressed in ovaries such as CU652878.

**Figure 7:**
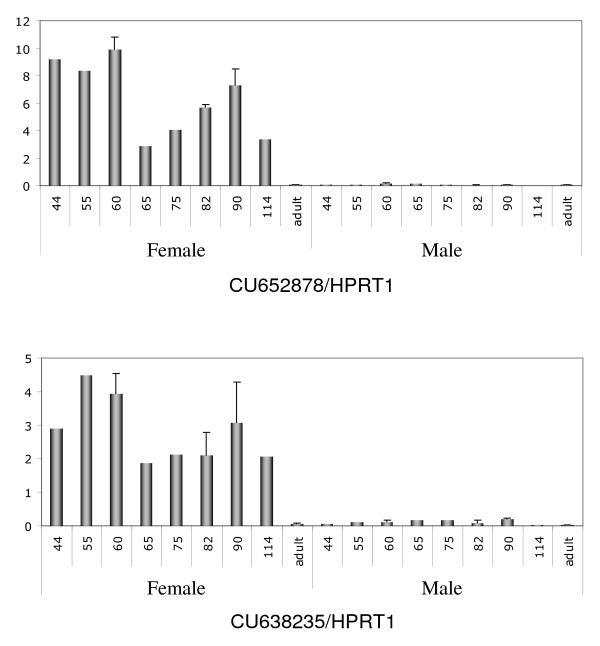
**Expression profiling of two unknown ovarian transcripts by real-time quantitative RT-PCR**. Quantification of CU652878 and CU638235 gene expression in sheep ovaries at Days 44, 55, 60, 65, 75, 82, 90 and 114 and adulthood. HPRT1 (hypoxanthine phosphoribosyltransferase) gene was used as a reporter gene. Both genes displayed female-specific gene expression. They were only detected during fetal life. Peak expression was observed at around D55 with a second peak at around D90, after which expression decreased and was not detected in adult ovary. SEM were calculated at stages D60, 82, 90 and adult.

Interestingly, 21 novel transcripts were also detected in the two libraries. Primers were designed for each of them [see Additional file 2] and RT-PCR analysis was performed using mini profiles (Fig. 6). The expression patterns of these 21 transcripts were classified into three categories: the first corresponded to transcripts expressed in both male and female gonads and somatic tissues, the second to transcripts specifically expressed in gonads of both sexes, and the third to those specifically expressed in the ovary (Fig. 6). Transcripts presenting a differential expression pattern between sexes or stages were subjected to real-time quantitative RT-PCR expression analysis (Fig. 7 and data not shown). This analysis showed that three transcripts were stage and sex -specifically expressed.

## Discussion

### Use of the SSH method to study meiosis and early folliculogenesis

Of the different strategies available to study transcriptome variations between two biological situations, Suppression Subtractive Hybridization (SSH) [43] is an efficient method to identify genes that are differentially regulated. Because it includes a normalization step, it enables the isolation of low abundance, differentially expressed transcripts. It does not require previous sequence knowledge, which is an advantage when the genome of the studied species has not been sequenced, and it can be started using PCR-amplified cDNAs. It is thus particularly well suited to biological situations such as embryonic or fetal development where specific genes are expressed and tiny amounts of RNA are available [44].

The present study aimed to analyze changes in the gene expression profile of sheep ovary during development using the SSH technique. The choice of SSH was based on the assumption that we would be able to discover factors expressed at lower levels than the genes classically detected by microarray techniques, particularly since commercial microarrays are only available for a small number of species that does not include sheep.

M and F cDNA-libraries were generated by SSH using mRNAs isolated from fetal ovaries at two developmental stages, and a total of 5,025 ESTs from both libraries were found to represent a maximum of 2,535 genes. The level of redundancy in the libraries was approximately 50%.

We used an approach based on the systematic sequencing of SSH products and gene analysis by functional categories. Out of the 2,101 uncommon ESTs that were cloned during this study, about 82% corresponded to contigs and singletons found to be unique, demonstrating the effectiveness of the experimental design utilized for these SSH libraries. Of the two libraries, the one dedicated to meiosis was of better quality than the F library, i.e. it contained more independent contigs (1578 versus 957), more non redundant contigs (1361/740) and more singletons (994/311).

In total, 2,101 non-redundant contigs were sequenced and analyzed. Nearly all of them presented a UniGene nucleotidic annotation (99%) and around two-thirds of the sequences were present in sheep genome databases, while one third presented homology with human or bovine genome databases. These results should be placed in context insofar as in the 99% of sequences with nucleic annotation, a large proportion displayed no protein annotation or possessed a hypothetical or unknown function. Major efforts regarding annotation are now necessary in order to improve our understanding of the functional role of these clones.

The cDNAs isolated from the M library included several transcripts that had previously been reported to be involved in the meiosis process [45]. Examples included *STK31 *[28], *SMC3 *[46], *DMRT7 *[47], *ZFP148 *[48]*MSH5 *[20], *MSH2 *[49], *Stathmin *[50], *DAZL*A [51], and *MUTL1 *[52]. Similar data had been observed regarding the F library, with the identification of *FOXO3A *[53], *SOHLH1 *[6] and *BMP4 *[54]. The detection of previously identified genes supported the validity of our experimental model and the use of SSH as the analytical method. Although the utilization of SSH in the current study was successful in identifying previously characterized genes, several expected genes were not present within the target cDNA library. Among these, *Stra8 *[1,24], *DMC1 *[18] for meiosis genes and *BMP15 *[55] and *Figla *[5], for folliculogenesis genes were not found within our subtracted libraries. The absence of these genes from the libraries may have been due to the use of PCR, and some of the cDNAs may not have been amplified as efficiently as others and thus have been lost from the SSH starting material. A similar inability to identify all expected known genes after SSH was recently reported by others and ascribed to incomplete representation of the total cDNA repertoire [31]. We can also suppose that the plating and sequencing of a larger number of clones could allow isolation of weakly expressed transcripts.

In addition, 1% of the transcripts thus isolated were unknown. They may correspond to additional exons of previously known genes, resulting from differential splicing specific to ovine species or to new genes not yet described (e.g. corresponding to non coding RNAs). Further 5' and 3'RACE analyses will be necessary to obtain full-length transcripts and to perform other sequence comparisons and functional analysis.

### Analysis of functional gene ontology

In order to understand the pathways involved in the initiation of prophase I meiosis and follicle formation, GO was used to cluster genes according to their biological function. The resulting classification mainly highlighted three biological processes, each representing more than 10% of the contigs taken into account: intracellular signaling cascade, cell surface receptors and DNA repair. These results should be modulated by the fact that only 25% of the contigs analyzed displayed GO annotation. However, this partial analysis was consistent with the fact that signaling molecules and DNA repair proteins are known to be important to regulating germ cell development and meiosis [56,57].

### Expressional analysis

RT-PCR analysis of three (STK31, MOV10L1, TEX11) of seven selected ESTs from the M library confirmed their differential expression between males and females. These three transcripts exhibited regulation in the sex, organ and stage from which they were cloned; i.e. female, ovary and meiosis prophase I stage. Real-time quantitative RT-PCR analyses of two of them (MOV10L1 [EMBL:CU654655] and TEX11 [EMBL:CU651916]) revealed that they were over-expressed in ovaries from days 55 to 82 and presented similar patterns to *DMC1*, a typical meiotic gene. This suggested that the M library contained ESTs representing genes differentially expressed in meiosis prophase 1 period. TEX11, initially described as being expressed during adult spermatogenesis, has recently been demonstrated on female meiotic chromosomes in mice [58], findings in agreement with our results. Moreover, it is noteworthy that *MOV10L1 *transcripts have never been described in female fetal gonads; they were previously described as being expressed in adult testis [45]. It is also interesting to note that the expression profile of the ovine Pecanex 1 gene differed from that described in the mouse [59]. It had previously been shown that the mouse Pecanex 1 transcript was only present in the germ line and not in the somatic cells of the testis, reaching its peak at the pachytene stage of meiotic prophase. However, in sheep, we detected Pecanex 1 in the fetal testis when male germ cells were not in meiosis.

The expression of unknown transcripts revealed that 20% of them were specific to gonads and 14% to ovary. Two of these unknown transcripts from the M library were more strongly expressed in the ovary and not expressed in the testis during fetal life. Their expression profiles were similar to those described in the goat for aromatase, activin β genes and 3β HSD [60], with a high level of transcripts at around D55 followed by a decline and then a further rise at around D75–82. This similarity of profiles suggests that these unknown genes may be involved in the regulation of steroid production. By refining our methods for sequence homology analysis, we found nucleotidic sequence homology between both CU652878 [EMBL:CU652878] and CU638235 [EMBL:CU638235] and two distinct bovine chromosome 6 genomic contigs (NW_001495163 and NW_001495159, respectively). No genes have been localized near these homologous regions.

This report also presents a medium scale sequencing of cDNAs representing genes in sheep. Indeed, of the 2,535 non-redundant ESTs selected from the M and F libraries, 771 were not reported in the Ovis aries EST database collection and therefore constituted an unique contribution to the Ovis aries EST pool. Though modest by comparison with databases for some other ruminants, such as cattle, our sequence collection for sheep may serve as a source for comparative studies in the bovids, an animal family of considerable importance to agronomic research.

## Conclusion

Using normalized cDNA subtraction as a transcript profiling tool to identify differentially expressed transcripts, we isolated several genes that may contribute toward understanding the mechanisms involved in meiosis and ovarian follicle differentiation in a mono-ovulatory species. Defects in these two processes are a leading cause of both infertility and birth defects. Further analyses of some selected genes in terms of their spatiotemporal expression patterns at the mRNA and protein levels in the developing ovarian follicle will be necessary to gain a clearer understanding of their functional roles.

In addition, 60 mer oligonucleotides from each of the 2101 unique ESTs will be designed and thus constitute a custom-made macroarray dedicated to ovine ovary differentiation. This tool will be used to evaluate modifications to gene expression under various nutritional and environmental conditions and to analyze their impacts on ovarian function.

## Methods

### Animals and tissue samples

Procedures for handling animals during this study were carried out in accordance with the INRA guidelines for the Care and Use of Agricultural Animals. The animals came from the INRA experimental farm at Brouessy, UCEA [61]. Cyclic Préalpes ewes were synchronized using an intravaginal progestogen sponge. Animals were inseminated at Day 0. Pregnant female tracts at different developmental stages were collected at slaughter and rapidly dissected to extract fetuses. Fetuses were collected on Days 44, 55, 60, 65, 75, 82, 90 and 114 of gestation. The gonads of adult animals were collected at slaughter. After puberty, gonads from both sexes were detached, then cut into small pieces and frozen immediately in liquid nitrogen (about 1/4 for the ovary and 1/20 for the testis). Other organs, such as the liver, kidney, brain and heart, were also collected, frozen immediately and then stored at -80°C.

### RNA extraction

Total RNA was extracted from each sample using TRIzol LS reagent (Invitrogen Life Technologies, Cergy-Pontoise, France), according to the method described by Chomczynski and Sacchi [62]. The quantitative yield of purification and the quality of RNAs were checked by spectrophotometrical scanning (Nanodrop ND1000 LABTECH) and by examination following 0.8% agarose gel electrophoresis to ensure equal purity and an absence of degradation in all samples.

### SSH experiments

One hundred micrograms of total RNA from each developmental stage (D55 and D82) were extracted, representing respectively the "meiosis" and "follicle formation" stages. PolyA+ mRNA were then fractionated from total RNA using the NucleoTrap mRNA Kit (BD Biosciences Clontech, Palo Alto, CA, USA) which combines a spin-column filter with oligo (dT) latex bead technology. Poly A^+ ^RNA was treated with 40 U of RNase-free DNaseI (Roche, Saclay, France) for 2 h at 37°C to prevent genomic DNA contamination. After phenol/chloroform extraction and ethanol precipitation, the DNase-treated poly A^+ ^RNA was resuspended in RNase-free water.

cDNAs were synthesized from 2 μg of polyA+ using 20 U AMV Reverse Transcriptase (BD Biosciences Clontech, Palo Alto, CA, USA) to generate the first cDNA-strand. Second-cDNA stands were produced with the DNA polymerase I (6 U/μl) (BD Biosciences Clontech, Palo Alto, CA, USA) provided in the kit. RsaI was added to these synthesized DS cDNA which were then incubated at 37°C. Tester and driver double strain cDNAs were prepared from each pool of gonads. According to the manufacturer's instructions, SSH experiments [43] were performed with the following steps: (i) *Rsa*I digestion of tester and driver cDNA; (ii) adaptor ligation (8 h at 68°C) to the testers: two tester populations were created with different adaptors (1 and 2R), driver cDNA had no adaptors; (iii) first hybridization: each tester cDNA was hybridized at 68°C overnight with an excess of the counterpart driver cDNA; (iv) second hybridization: the 2 samples from the first hybridization were newly hybridized with fresh driver cDNA; (v) two consecutive PCR amplification steps with primers designed according to the adaptor sequence were performed in order to enrich the differentially expressed sequences and reduce background. The first comprised 27 cycles (94°C for 30 sec, 66°C for 30 sec, 72°C for 1 min 30 sec) using the end of the adaptors as primers. The second PCR comprised 10 or 12 cycles (F and M libraries, respectively) using the nested primers (included in adaptors 1 and 2R) (94°C for 30 sec, 68°C for 30 sec, 72°C for 1 min 30 sec). All the primers used during SSH experiments are listed in additional file 3 [see Additional file 3].

The efficiency of subtraction was analyzed using PCR by comparison with the abundance of cDNAs before and after subtraction on the constitutively expressed gene G3PDH (94°C for 30 sec, 60°C for 30 sec, 72°C for 1 min 30 sec).

Five microliters aliquots were collected from 50-μl PCR reactions after 18, 23, 28, 33 and 38 cycles. Amplification was quantified by densitometry. The efficiency of subtraction was confirmed by comparing the abundance of cDNAs before and after subtraction using PCR with bovine GAPDH [see Additional file 4].

### cDNA cloning, sequencing, data analysis and dbEST submission

The subtracted PCR products generated by SSH were purified using the GFX kit (GE Healthcare, Orsay, France), cloned into the pGEMT easy vector (Promega France, Charbonnière les Bains, France) and then transformed into *Escherichia coli *DH5-alpha T1 Max Efficiency Cells (Invitrogen, Cergy-Pontoise, France). Positive clones were picked out according to blue/white selection and 7296 colonies were transferred robotically into 384 well plates at the INRA National Biological Resources Centre for Animal Genomics [63], where they were grown in 2xYT medium overnight at 37°C. All individual colonies were spotted on nylon membrane and then fixed with NaOH, neutralized by Tris-HCl and stored at 4°C prior to hybridization.

Clones were randomly selected and single-pass sequenced with a vector primer from sequencing companies (Genome Express, [64] and MWG, [65]). On average, 500 clones were sequenced and then hybridized on the macro-membrane containing the libraries in order to identify any redundant clones. After hybridization, another batch of 500 clones was sequenced, hybridized and so on.

Using SIGENAE bioinformatics facilities [41], EST quality was computed using a home-made workflow system and the ESTs were then cleaned of vector and primer sequences and sequences containing contaminants such as E. Coli, Yeast, Mitochondria, Ribosome. SSH cDNA libraries usually contained insertion sizes within a range of 200–800 bp. A sequence was only selected for further analysis if it had a valid status, i.e. containing at least 100 bp outside repeats with a phred score over 20, without any vectors and adaptors so as to prevent chimerical contig linking sequences, for instance because of unclipped vectors.

Contigs and EST singletons of SIGENAE Sheep clustering 5 [41] were annotated for sequence homologies by running BLASTX or BLASTN against the following databases for different mammalian species (human, bovine, ovine): UniProtKB/Swiss-Prot Release 53.0 of 29-May-2007, UniProtKB/TrEMBL Release 36.0 of 29-May-2007, ProDom Release CG67, Sigenae Sheep Contigs V4, TIGR Cattle BtGI 12, UniGene Cattle Build #84, UniGene Human Build #202, UniGene Sheep Build #11, Ensembl Cattle Transcripts Btau_3.1 44 and Ensembl Human Transcripts NCBI36 44.

Only sequences with a PHRED score over 20 on at least 100 bp were released in the EST division of the EMBL-EBI (European Molecular Biology Laboratory – European Bioinformatics Institute) Nucleotide Sequence Database with accession numbers.

Accession numbers in the EMBL Nucleotide Sequence Database ran from CU637754 to CU638663 and from CU651679 to CU655811, except for CU652171, CU652212, CU652215, CU652218, CU652484, CU652528, CU652531, CU652642, CU652698, CU652701, CU652704, CU652776, CU652780, CU652834, CU652837, CU652840, CU652843 and CU652893.

### Contig similarity search and functional assignments

Similarity searches of each contig sequence were performed using BLASTX or BLASTN programs with different cut-off e-value criteria, depending on the databases used. These e-values were 1e-02 with the ProDom, TIGR and UniGene databases; 1e-05 against UniProt databases; 1e-10 against Ensembl transcripts and 1e-30 against other SIGENAE contigs. Functional annotation was carried out using Gene Ontology [66]. Next, the GO categorization scheme of classification by biological process, cellular component and molecular function was used to generate representative pie diagrams [67].

### Semi-quantitative reverse transcription-PCR (Semi-quantitative RT-PCR)

Total RNA was isolated from the ovaries, testis, liver, heart, brain and kidney of fetal or adult origin using the TRIzol LS reagent (Invitrogen Life Technologies, Cergy-Pontoise, France) according to the manufacturer's instructions. RNA extraction, DNase treatment, and cDNA synthesis were carried out as previously described [37]. Briefly, 1 μl (corresponding to 0.25 μg reverse transcribed total RNA) of each RT mix was amplified in 25 μl PCR using 0.5 U Taq polymerase (TaKaRa, Lonza, Verviers, Belgium), 200 μM of each dNTP, and 150 nM of each primer. The sequences of the primers used and the PCR conditions utilized are shown in additional files 2 and 5 [see additional file 2], [see additional file 5].

The GAPDH gene was used as a cDNA quantity control. PCR conditions were as follows: 25 cycles of 94°C for 30 sec, 58°C for 30 s and 72°C for 30 sec, followed by a single cycle at 72°C for 10 min.

### Real-time quantitative RT-PCR of differentially expressed transcripts

cDNA (50 ng) from ovary and testis was obtained by the reverse transcription of 5 μg RNA for each sample using Superscript II (Invitrogen, Cergy-Pontoise, France) and mixed with 12.5 μl (1×) of ABsolute blue QPCR SYBR Green ROX mix (Abgene, Les Ulis, France), 300 nM from each primer [see Additional file 6], in a total volume of 25 μl. The reaction mixture was finally transferred into a 96-well optical reaction plate, sealed with appropriate optical caps, and run on the ABI Prism 7700 HT apparatus (Applied Biosystems). Standard controls of both the specificity and efficiency of the qPCR assays were performed. All expression data were normalized to the HPRT1 expression level. The concentration of the target genes were divided by the concentration of the HPRT1 gene thus showing the relative expression of the mRNA in the tissue. This calculation was performed for all stages analyzed. The sequences of the primers used, and the qPCR conditions prevailing, are shown in additional file 6 [see Additional file 6]. All qPCR experiments are made in duplicate and for some stages (D60, 82, 90 and adult), different animals, RNA extractions, and RT were used. SEM were calculated when two independent qPCR from 2 different biological materials were done.

## Competing interests

The authors declare that they have no competing interests.

## Authors' contributions

AB carried out screening procedures, conducted all expression validations, performed gene ontology annotations and helped to draft the manuscript. BMP participated in design of the study, carried out gonad dissections, mRNA extractions, constructed the SSH and helped to draft the manuscript. CCa carried out sequence and bioinformatics analyses. EPo participated in data analysis and real-time quantitative RT-PCR experiments; EPa participated in data analysis and interpretation of the results. CCo conceived the study, supervised the experiments and helped to write the manuscript. All authors read and approved the final manuscript.

## Supplementary Material

Additional file 1 List of genes found more than 10 times in both libraries. The table provided shows the listing of genes represented 10 times or more in M and F libraries. Click here for file

Additional file 2 PCR primer sequences of unknown clones and experimental conditions. This table provided sequences and experimental conditions of PCR primer amplified the unknown clones. Click here for file

Additional file 3 List of primers used during SSH experiments. This table provided lists the primers used during SSH and their sequences. Click here for file

Additional file 4 Evaluation of subtraction efficiency. SSH subtraction efficiency was determined by analyzing the amount of GAPDH present in both the unsubtracted starting cDNA and subtracted target cDNA through the use of increasing numbers of PCR cycles. In both libraries, the GAPDH cDNA fragment was absent of the subtracted samples. In the unsubtracted samples the amplified band was observed only following 18 PCR cycles. Click here for file

Additional file 5 Sequences of PCR primers and conditions for mini profiles. This table provided sequences and experimental conditions of the PCR primers used for mini profile procedure *i.e*. from male and female gonads at 55 and 82 days post coitum. Click here for file

Additional file 6 Sequences of qPCR primers and experimental conditions. The table shows the sequence and experimental conditions of the primers used for qPCR. Click here for file
