# Supplementary material for: Identification of transcripts involved in meiosis and follicle formation during ovine ovary development
Source: BMC Genomics. 2008 Sep 23;9:436. doi: 10.1186/1471-2164-9-436 (PMC2566313; doi:10.1186/1471-2164-9-436)
Supplement: Additional file 1 — List of genes found more than 10 times in both libraries. The table provided shows the listing of genes represented 10 times or more in M and F libraries. [file 1471-2164-9-436-S1.doc]

**Additional file 1: List of genes found more than 10 times in both libraries**

| **Libraries** | **Times** | **Genes (OMIM number)** |
| --- | --- | --- |
| M | 12 | Bos taurus hypothetical protein, LOC783526 |
| M | 13 | Cytochrome c oxidase subunit 3 |
| F | 10 | Proteasome subunit, alpha type, 2 (176842) |
| F | 10 | Luc7-Like (607782) |
| F | 10 | Actin, alpha (102610) |
| F | 11 | Ribosomal protein S10 (603632) |
| F | 11 | Bos taurus hypothetical protein, LOC504658 |
| F | 11 | Similar to PRKC apoptosis WT1 regulator or PAWR, LOC532789 |
| F | 12 | Coiled-coil-helix-coiled-coil-helix domain-containing protein 5 or CHCHD5 |
| F | 13 | Myosin binding protein C, slow type (160794) |
| F | 14 | Translin (600575) |
| F | 15 | Decorin (125255) |
| F | 17 | mitochondrion, complete genome |
