# Supplementary material for: Identification of transcripts involved in meiosis and follicle formation during ovine ovary development
Source: BMC Genomics. 2008 Sep 23;9:436. doi: 10.1186/1471-2164-9-436 (PMC2566313; doi:10.1186/1471-2164-9-436)
Supplement: Additional file 2 — PCR primer sequences of unknown clones and experimental conditions. This table provided sequences and experimental conditions of PCR primer amplified the unknown clones. [file 1471-2164-9-436-S2.doc]

**Additional file 2: PCR primer sequences of unknown clones and experimental conditions.**

| **Gene** | **Primers** | **Annealing temperature (°C)** | **Elongation time (sec)** | **Cycles** | **MgCl_2_ (mM)** | **Other** |
| --- | --- | --- | --- | --- | --- | --- |
| CU652348 | 5'-CCTTTGGGACTGAACAATGG-3' | 56°C | 30 | 35 | 2.5 |  |
|  | 5'-ACTCAATCCATCGGATGCTC-3' |  |  |  |  |  |
| CU637818 | 5'-TCACTGCAGGGGTCAAAATG-3' | 56°C | 30 | 29 | 2.5 |  |
|  | 5'-GGCTTCTGCAACACAGTGAA-3' |  |  |  |  |  |
| CU655172 | 5'-ACCCCAAATTGGTAGGGTTT-3' | 55°C | 30 | 35 | 2.5 | 2% DMSO |
|  | 5'-CGCTGGGTCTAATGCTCAAT-3' |  |  |  |  |  |
| CU652735 | 5'-CTGCTTTGCAAATCCAGTCA-3' | 55°C | 30 | 35 | 2.5 |  |
|  | 5'-CTGTGGCCTTTTGGTTGATT-3' |  |  |  |  |  |
| CU638285 | 5'-AGCACCGCATACCAATTTTC-3' | 57°C | 30 | 30 | 2.5 |  |
|  | 5'-GTCCAGGTGCTCATTTGTTC-3' |  |  |  |  |  |
| CU652180 | 5'-CTTTGGTTTGCTGTTGCTCA-3' | 55°C | 30 | 35 | 2.5 |  |
|  | 5'-AAAGTGCCTTCCTGGTCAGA-3' |  |  |  |  |  |
| CU652878 | 5'-AGGAAGCATTTTGTGGGTTG-3' | 56°C | 30 | 35 | 2.5 |  |
|  | 5'-CTCCTTGCCCAGTGGATAAA-3' |  |  |  |  |  |
| CU652467 | 5'-TCCCCTGTCATCCATTGTTT-3' | 55°C | 30 | 35 | 2.5 |  |
|  | 5'-GCTCATGCTCAAATGCTGAA-3' |  |  |  |  |  |
| CU638623 | 5’-TAGTAAGGACTCCCTATGGC-3’ | 56°C | 30 | 35 | 2.5 |  |
|  | 5’-TAGACTCCTGTGAATGGTGG-3’ |  |  |  |  |  |
| CU637992 | 5’-GTTGTGGCTTATAATACACG-3’ | 52°C | 30 | 35 | 2.5 |  |
|  | 5’-GATTTCAAATACCGACAAGG-3’ |  |  |  |  |  |
| CU655510 | 5’-ATGCCAGCGTCTGGTTTAAG-3’ | 57°C | 30 | 35 | 2.5 |  |
|  | 5’-CAGCCTGCATCTGTCAGCTA-3’ |  |  |  |  |  |
| CU652595 | 5'-GACAAATGAAAAGGCCCAGA-3' | 55°C | 30 | 35 | 2.5 |  |
|  | 5'-CTCAGTCTCCCAGGCCATAA-3' |  |  |  |  |  |
| CU637945 | 5'-ACGTGCATGTTTGAGTCAGG-3' | 56°C | 30 | 35 | 2.5 |  |
|  | 5'-AGGGTATTTTCGGGGAACAC-3' |  |  |  |  |  |
| CU653723 | 5'-AAGGAGCCTCCAGGAAGAAG-3' | 58°C | 30 | 35 | 2.5 |  |
|  | 5'-CCTCACTCATGCAATTGTGG-3' |  |  |  |  |  |
| CU654447 | 5'-ACTGACCACACCATGTGGAA-3' | 57°C | 30 | 35 | 2,5 | 2% formamide |
|  | 5'-CCCATGGTGTCAGCTACTTG-3' |  |  |  |  |  |
| CU638013 | 5'-CATGTGTTGGAGTTGTGTGG-3' | 57°C | 30 | 35 | 2.5 |  |
|  | 5'-GGTAGAACAGGACAGTGAGG-3' |  |  |  |  |  |
| CU638235 | 5'-GATGACCTGCCATTTCCAGT-3' | 56°C | 30 | 30 | 2.5 |  |
|  | 5'-CTTCCTGAAAGGCAAAGTGG-3' |  |  |  |  |  |
| CU654856 | 5'-GATTGTTTCCTCACGGGTTC-3' | 57°C | 30 | 35 | 2.5 |  |
|  | 5'-AACTCACCGGAGACTGCAAC-3' |  |  |  |  |  |
| CU652952 | 5'-TTTATTGGTGGGCTGTGTCA-3' | 56°C | 30 | 35 | 2.5 |  |
|  | 5'-AGCCAGGAGGTCTTGGAAAT-3' |  |  |  |  |  |
| CU654716 | 5'-ACTTCAGACCGCACACTTCC-3' | 57°C | 30 | 30 | 2.5 |  |
|  | 5'-GTGTCGCTGAGGACCATTTT-3' |  |  |  |  |  |
| CU654342 | 5’-AAAACCCAGTGTTTCCTTCC-3’ | 54°C | 30 | 35 | 2.5 |  |
|  | 5’-ACTATCAACTGTATGCCGTC-3’ |  |  |  |  |  |
