# Supplementary material for: Identification of transcripts involved in meiosis and follicle formation during ovine ovary development
Source: BMC Genomics. 2008 Sep 23;9:436. doi: 10.1186/1471-2164-9-436 (PMC2566313; doi:10.1186/1471-2164-9-436)
Supplement: Additional file 3 — List of primers used during SSH experiments. This table provided lists the primers used during SSH and their sequences. [file 1471-2164-9-436-S3.doc]

**Additional file 3: List of primers used during SSH experiments**

| **Primer** | **Sequence** |
| --- | --- |
| Adaptor 1 | 5’-CTAATACGACTCACTATAGGGCTCGAGCGGCCGCCCGGGCAGGT-3’ |
| Adaptor 2R | 5’-CTAATACGACTCACTATAGGGCAGCGTGGTCGCGGCCGAGGT-3’ |
| Nested primer 1 | 5’-TCGAGCGGCCGCCCGGGCAGGT-3’ |
| Nested primer 2R | 5’-AGCGTGGTCGCGGCCGAGGT-3’ |
| GAPDH up | 5’-CCTGGCCAAGGTCATCCATG-3’ |
| GAPDH dw | 5’-TTGAGGGCAATGCCAGCCCC-3’ |
