# Supplementary material for: Identification of transcripts involved in meiosis and follicle formation during ovine ovary development
Source: BMC Genomics. 2008 Sep 23;9:436. doi: 10.1186/1471-2164-9-436 (PMC2566313; doi:10.1186/1471-2164-9-436)

Additional file 4 – Evaluation of subtraction efficiency

SSH subtraction efficiency was determined by analyzing the amount of GAPDH (housekeeping gene) present in both the unsubtracted starting cDNA and subtracted target cDNA through the use of increasing numbers of PCR cycles. In both libraries, the GAPDH cDNA fragment (435bp) was absent of the subtracted samples. In the unsubtracted samples the amplified band was observed only following 18 PCR cycles.

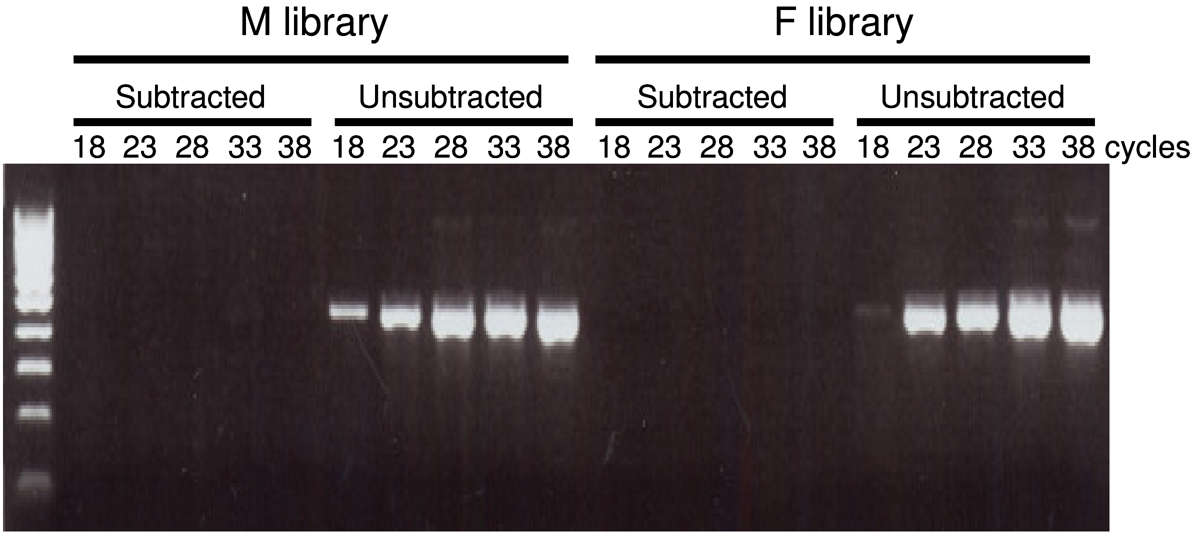

Supplement: Additional file 4 — Evaluation of subtraction efficiency. SSH subtraction efficiency was determined by analyzing the amount of GAPDH present in both the unsubtracted starting cDNA and subtracted target cDNA through the use of increasing numbers of PCR cycles. In both libraries, the GAPDH cDNA fragment was absent of the subtracted samples. In the unsubtracted samples the amplified band was observed only following 18 PCR cycles. [file 1471-2164-9-436-S4.pdf]
