# Supplementary material for: Identification of transcripts involved in meiosis and follicle formation during ovine ovary development
Source: BMC Genomics. 2008 Sep 23;9:436. doi: 10.1186/1471-2164-9-436 (PMC2566313; doi:10.1186/1471-2164-9-436)
Supplement: Additional file 5 — Sequences of PCR primers and conditions for mini profiles. This table provided sequences and experimental conditions of the PCR primers used for mini profile procedure i.e. from male and female gonads at 55 and 82 days post coitum. [file 1471-2164-9-436-S5.doc]

**Additional file 5: Sequences of PCR primers and conditions for mini profiles.**

| **Gene** | **Primers** | **Annealing temperature (°C)** | **Elongation time (sec)** | **Cycles** | **MgCl_2_ (mM)** |
| --- | --- | --- | --- | --- | --- |
| **DMC1** | 5'-GCCGATCCAGGAGCAACTAT-3' | 58 | 30 | 30 | 1,5 |
|  | 5'-CCACCTACTCCTTGGCATCCC-3' |  |  |  |  |
| **GAPDH** | 5'-AGGCCATCACCATCTTCCAG-3' | 58 | 30 | 22 | 1,5 |
|  | 5'-GGCGTGGACAGTGGTCATAA-3' |  |  |  |  |
| **Maelstrom** | 5'-GCCAGCAATGGTGTGACACC-3' | 58 | 30 | 30 | 2,5 |
| CU637892 | 5'-CGGGTAATCCCTCTTCCCCG-3' |  |  |  |  |
| **MOV10L1** | 5'-GGTACTGCAGTGATTATGGC-3' | 56 | 30 | 35 | 2,5 |
| CU654655 | 5'-AACCTTCACACACACTCTCC-3' |  |  |  |  |
| **Pecanex** | 5'-GCTAGCTAATGAGACGATGC-3' | 56 | 30 | 30 | 2,5 |
| CU655519 | 5'-AGCAAAGATCCATTCGTCCC-3' |  |  |  |  |
| **STK31** | 5'-GTGTGGATCACTTGCTATCC-3' | 56 | 30 | 35 | 2,5 |
| CU655270 | 5'-TATCAAAGGACGCTTGCTGC-3' |  |  |  |  |
| **TEX11** | 5'-CAGAGACATCTGGAATGTCC-3' | 56 | 30 | 32 | 2,5 |
| CU651916 | 5'-TCATCAGACAGAGAACCTCC-3' |  |  |  |  |
| **TEX14** | 5'-AGCACGAAGCCAGAGAGAAG-3' | 59 | 30 | 30 | 2,5 |
| CU638174 | 5'-ACAGACCCACTGCCCTAATG-3' |  |  |  |  |
| **ZFP148** | 5'-CATCCTCATCAGATAAAGCC-3' | 55 | 30 | 35 | 2,5 |
| CU655301 | 5'-TCATTTGGTAAGCTTGGTGG-3' |  |  |  |  |
