# Supplementary material for: Identification of transcripts involved in meiosis and follicle formation during ovine ovary development
Source: BMC Genomics. 2008 Sep 23;9:436. doi: 10.1186/1471-2164-9-436 (PMC2566313; doi:10.1186/1471-2164-9-436)
Supplement: Additional file 6 — Sequences of qPCR primers and experimental conditions. The table shows the sequence and experimental conditions of the primers used for qPCR. [file 1471-2164-9-436-S6.doc]

**Additional file 6: Sequences of qPCR primers and experimental conditions.**

Oligonucleotide primers were designed for selected genes using the PrimerExpress Designer software (Perkin Elmer).

| **Gene** | **Primers** |
| --- | --- |
| **CU652878** | 5'-GAAGTTGGGCTTTGAAAGACCA-3' |
|  | 5'-CAAATTGCTGGAGTTCAAACCC-3' |
| **CU638235** | 5'-ATGCAAAATGAAAATCCTCTTGCT-3' |
|  | 5'-TGGAAACGGCAGGTCATCATC-3' |
| **DMC1** | 5'-TCATACCCTCTGTGTGACAGCTC-3' |
|  | 5'-GGCGATCTGGACGGAAAGTA-3' |
| **HPRT1** | 5'-TGGACTAATTATGGACAGGACCG-3' |
|  | 5'-TATAGCCCCCCTTGAGCACA-3' |
| **TEX11** | 5'-AGAGACATCTGGAATCTCCTGAAAA-3' |
|  | 5'-TCCAGTAATGGGTCACTCAGTTTG-3' |
